# Tsallis Entropy Index *q* and the Complexity Measure of Seismicity in Natural Time under Time Reversal before the M9 Tohoku Earthquake in 2011

**DOI:** 10.3390/e20100757

**Published:** 2018-10-02

**Authors:** Panayiotis A. Varotsos, Nicholas V. Sarlis, Efthimios S. Skordas

**Affiliations:** 1 Section of Solid State Physics, Department of Physics, National and Kapodistrian University of Athens, Panepistimiopolis, Zografos, 157 84 Athens, Greece; 2 Solid Earth Physics Institute, Department of Physics, National and Kapodistrian University of Athens, Panepistimiopolis, Zografos, 157 84 Athens, Greece

**Keywords:** earthquakes, natural time analysis, non-extensive statistical mechanics, index *q*

## Abstract

The observed earthquake scaling laws indicate the existence of phenomena closely associated with the proximity of the system to a critical point. Taking this view that earthquakes are critical phenomena (dynamic phase transitions), here we investigate whether in this case the Lifshitz–Slyozov–Wagner (LSW) theory for phase transitions showing that the characteristic size of the minority phase droplets grows with time as $t^{1/3}$ is applicable. To achieve this goal, we analyzed the Japanese seismic data in a new time domain termed natural time and find that an LSW behavior is actually obeyed by a precursory change of seismicity and in particular by the fluctuations of the entropy change of seismicity under time reversal before the Tohoku earthquake of magnitude 9.0 that occurred on 11 March 2011 in Japan. Furthermore, the Tsallis entropic index *q* is found to exhibit a precursory increase.

## 1. Introduction

Earthquakes exhibit complex correlations in time, space and magnitude and have been the object of a multitude of studies [1,2,3,4,5,6,7]. The observed earthquake scaling laws (e.g., [8]) are considered to indicate the existence of phenomena closely associated with the proximity of the system to a critical point (e.g., [9]). Accepting this view that earthquakes are critical phenomena (dynamic phase transitions, where a mainshock is the new phase), the quantity by which one can identify the approach of a dynamical system to the state of criticality is termed order parameter. This parameter in the frame of a new time domain termed natural time χ [10] is just the quantity $\kappa_{1}=\langle \chi^{2}\rangle -{\langle \chi \rangle }^{2}$, as explained in Section 2 (cf. pp. 249–254 of Ref. [11]). The following two key properties have been shown [10,11] for the probability density function (PDF) $P(\kappa_{1})$ of the $\kappa_{1}$ values in an earthquake catalog: First, different seismic areas $\sigma P(\kappa_{1})$ versus $(\mu -\kappa_{1})/\sigma$—where μ stands for the mean of the $\kappa_{1}$ values and σ for their standard deviation—fall on a non-Gaussian universal curve which has a left exponential tail, showing that an extreme fluctuation may be orders of magnitude more probable than it would be if Gaussianity was valid, pointing to the existence of extreme events. Second, the PDF $P(\kappa_{1})$ versus $\kappa_{1}$ before strong earthquakes exhibits a bimodal feature. This, for example, has been observed before the M7.3 Landers and before the M7.1 Hector Mine earthquakes that occurred in Southern California in 1992 and 1999, respectively (see pp. 274 and 278 of Ref. [11]). Another very recent example studied in Ref. [12] is the case of the M8.2 earthquake that occurred in the Chiapas region in Mexico on 7 September 2017 to which we now turn.

In the case of Mexico, the seismicity has been studied in natural time in Ref. [13] in the six tectonic regions Baja California (BC), Jalisco-Colima (J), Michoacán(M), Guerrero (G), Oaxaca (O) and Chiapas (CH) of the Mexican Pacific Coast shown in Figure 2 of Ref. [12]. This study showed that only for earthquakes in the regions CH, G and O a bimodal feature appears in the PDF $P(\kappa_{1})$ versus $\kappa_{1}$ (see their Figure 3a). Among these three regions, the first one, i.e., CH, the PDF $P(\kappa_{1})$ vs $\kappa_{1}$ of which is shown in Figure 3 of Ref. [12], had the highest probability for an extreme fluctuation (large earthquake) as can be seen by comparing their left exponential tail of the $\sigma P(\kappa_{1})$ versus $(\mu -\kappa_{1})/\sigma$ plot depicted in Figure 4 of Ref. [12], where the results of all six regions are shown. These results reveal that in principle extreme events in the Chiapas region were expected from the natural time analysis, as actually happened upon the occurrence of the M8.2 earthquake in this region.

In addition, by employing natural time analysis in Ref. [12], it was found that the entropy change ΔS under time reversal of the seismicity in the Chiapas area exhibited a clear minimum on 14 June 2017, which signaled that a major event was impending there as actually happened almost three months later with the occurrence of the M8.2 earthquake on 7 September 2017. This is so, because ΔS constitutes a key measure that may identify when the system approaches the critical point (dynamic phase transition) [11]. For example, ΔS has been applied [14] for the identification of the impending sudden cardiac death risk. Furthermore, it has been shown that ΔS provides a useful tool [15] to investigate the predictability of the Olami–Feder–Christensen (OFC) model for earthquakes [16], which is probably [17] the most studied non-conservative self-organized criticality model. In particular, it was found that ΔS exhibits a clear minimum [11] (or maximum if we define [15] $\Delta S\equiv S_{-}-S$ instead of $\Delta S\equiv S-S_{-}$ used here, see Section 2) before a large avalanche in the OFC model, which corresponds to a large earthquake. Furthermore, in a more recent study [18], by analyzing the seismicity during the six-year period 2012–2017 in natural time in the Chiapas region where the M8.2 earthquake occurred, we found that, on the same date as above, i.e., 14 June 2017, the complexity measure $\Lambda_{i}$ (see Section 2) associated with the fluctuations of the entropy change under time reversal exhibits an abrupt increase along with a simultaneous increase of the Tsallis entropic index *q* [19,20,21,22,23,24].The temporal variations of the latter entropic index before strong earthquakes arouse an intense international interest and has been studied by several workers [25,26,27,28,29,30,31,32].

Recapitulating the aforementioned recent studies [12] related with the M8.2 earthquake that occurred in Mexico on 7 September 2017, which is Mexico’s largest earthquake in more than a century, we can say that upon employing natural time analysis we found that almost three months before its occurrence the following precursory behavior was identified: The entropy change under time reversal exhibits a minimum [12] along with increased fluctuations of the entropy change under time reversal as well as by a simultaneous increase of the Tsallis entropic index *q* [18]. It is the main scope of this paper to investigate whether precursory behavior existed also before the super-giant M9 Tohoku earthquake that occurred in Japan on 11 March 2011. In addition, here we investigate whether the seminal work in 1961 by Lifshitz and Slyozov [33] and independently by Wagner [34], on phase transitions is applicable to this super-giant earthquake which can be considered, as mentioned above in the first paragraph, as a dynamic phase transition. We stress, however, that the present work differs essentially from our previous recent studies related with the M8.2 earthquake in Mexico, because—beyond the significant difference as far as their magnitudes is concerned—the following holds: These recent studies on Mexico’s earthquake have been carried out by analyzing in natural time the seismicity in Chiapas region during 2012–2017 (where the M8.2 earthquake occurred) since the aforementioned work in 2013 by Ramírez-Rojas and Flores-Márquez, which analyzed in natural time the seismicity in the six tectonic regions of Mexico, forwarded arguments that in the Chiapas region extreme events (i.e., large earthquakes) have been expected [13]. Unfortunately, such a work providing an estimate of the region where the M9 Tohoku earthquake was going to occur had not been published before its occurrence in 2011. (Such an estimate of the epicentral area was published recently [35] as explained below in Section 5.) In view of the lack of such an information at that time, natural time analysis is made here for the seismicity that occurred in the whole Japanese area $N_{25}^{46}E_{125}^{148}$ for the period 1 January 1984–11 March 2011, the day of the M9 Tohoku earthquake. In other words, here we are going to answer the following question: If a super-giant earthquake is going to occur in a large area such as Japan, is it possible to identify a precursory behavior and estimate when the system approaches the critical point (earthquake occurrence)? This is answered in light of the fact that it is nowadays widely accepted, as for example stated by Holliday et al. [9], that the observed scaling laws indicate the existence of phenomena closely associated with the proximity of the system to a critical point as already mentioned. In particular, here we mainly focus our investigation on the complexity measure $\Lambda_{i}$ associated with the fluctuations of the entropy change under time reversal and on the Tsallis entropic index *q* which still continues to arouse international interest [36] by analyzing in natural time the seismicity occurring allover that area.

This paper is structured as follows: A summary of natural time analysis is given in Section 2, while the data along with the procedure for their analysis are presented in Section 3. Our results are described in Section 4 and a discussion follows in Section 5. Finally, our conclusions are summarized in Section 6.

## 2. Natural Time Analysis Background

Natural time analysis, introduced in the beginning of the 2000s [37,38,39,40,41], uncovers unique dynamic features hidden behind the time series of complex systems. For seismicity, in a time series comprising *N* earthquakes, the natural time $\chi_{k}=k/N$ serves as an index for the occurrence of the *k*th earthquake. This index together with the energy $Q_{k}$ released during the *k*th earthquake of magnitude $M_{k}$, i.e., the pair $\left(\chi_{k},Q_{k}\right)$, is studied in natural time analysis. Equivalently, one studies the pair $\left(\chi_{k},p_{k}\right)$, where

(1) $$p_{k}=\frac{Q_{k}}{\sum_{n=1}^{N}Q_{n}}$$

stands for the normalized energy released during the *k*th earthquake. The variance of χ weighted for $p_{k}$, labeled by $\kappa_{1}$, is given by [10,11,37,40,41,42]

(2) $$\kappa_{1}=\sum_{k=1}^{N}p_{k}{\left(\chi_{k}\right)}^{2}-{\left(\sum_{k=1}^{N}p_{k}\chi_{k}\right)}^{2}$$

where $Q_{k}$, and hence $p_{k}$, for earthquakes is estimated through the usual relation [43]

(3) $$Q_{k}\propto 10^{1.5M_{k}}$$

In natural time analysis, an order parameter for seismicity has been proposed. In particular, it has been explained in Ref. [10] (see also pp. 249–253 of Ref. [11]) that the quantity $\kappa_{1}$ given by Equation (2) can be considered as an order parameter for seismicity since it changes abruptly when a mainshock (the new phase) occurs, and in addition the feature of its fluctuations resemble those in other non-equilibrium and equilibrium critical systems. Note that at least six earthquakes are needed for obtaining reliable $\kappa_{1}$ [10]. It has been found that the quantity $\kappa_{1}$ is a key parameter that enables recognition of the complex dynamical system under study entering the critical stage [11,37,38,39]. In short, Varotsos et al. [44] (see also p. 343 of Ref. [11]) showed that $\kappa_{1}$ becomes equal to 0.070 at the critical state for a variety of dynamical models. In Table 8.1 of Ref. [11], one can find a compilation of 14 cases in which the condition $\kappa_{1}$ = 0.070 has been ascertained. (This was also later confirmed in the analyses of very recent experimental results in Japan by Hayakawa and coworkers [45,46].) In addition, natural time has been recently employed by Turcotte and coworkers [47,48,49,50] as a basis for a new method to estimate the current level of seismic risk.

The entropy *S* in natural time is defined as the derivative with respect to *d* of the fluctuation function $\langle \chi^{d}\rangle -{\langle \chi \rangle }^{d}$ at d=1, which results in:

(4) S≡〈χlnχ〉−〈χ〉ln〈χ〉

where $\langle f\left(\chi \right)\rangle =\sum_{k=1}^{N}p_{k}f\left(\chi_{k}\right)$. It is dynamic entropy and its value upon considering the time reversal *T*, i.e., $Tp_{k}=p_{N-k+1}$, is labeled by $S_{-}$. The value of $S_{-}$ is, in general, different from *S* (e.g., [14]) (see also Ref. [51] and references therein), and thus *S* satisfies the conditions to be “causal”. The physical meaning of the change of entropy $\Delta S\equiv S-S_{-}$ in natural time under time reversal has been discussed in Refs. [11,14,51]. The entropy *S* is a dynamic entropy, as mentioned, that exhibits [52] concavity, positivity, and Lesche stability [53,54].

Using a moving window of length *i* (number of consecutive events) sliding through the time series of *L* consecutive earthquakes the entropy in natural time has been determined for each position j=1,2,…,L−i of the sliding window. Thus, a time series of $S_{i}$ is obtained. By considering the standard deviation $\sigma (\Delta S_{i})$ of the time series of $\Delta S_{i}\equiv S_{i}-{\left(S_{-}\right)}_{i}$, we define [11,18,55] the complexity measure $\Lambda_{i}$

(5) $$\Lambda_{i}=\frac{\sigma (\Delta S_{i})}{\sigma (\Delta S_{100})}$$

when a moving window of *i* consecutive events is sliding through the time series and the denominator has been selected [18] to correspond to the standard deviation $\sigma (\Delta S_{100})$ of the time series of $\Delta S_{i}$ of *i* = 100 events. This complexity measure quantifies how the statistics of $\Delta S_{i}$ time series changes upon increasing the scale from 100 events to a longer scale, e.g., $i=10^{3}$ events. The calculations are carried out by means of a window of length *i* (= number of successive earthquakes) sliding, each time by one earthquake, through the whole time series. The entropies *S* and $S_{-}$, and therefrom their difference $\Delta S_{i}$, are calculated each time, thus we also form a new time series consisting of successive $\Delta S_{i}$ values and the complexity measure $\Lambda_{i}$ is determined according to its definition given in Equation (5).

## 3. Data and Analysis

The Japan Meteorological Agency (JMA) seismic catalogue was used (e.g., see Refs. [35,56]). We considered all earthquakes of magnitude M ≥ 3.5 from 1984 until the Tohoku earthquake occurrence on 11 March 2011 within the area 25${}^{\circ }$–46${}^{\circ }$ N, 125${}^{\circ }$–148${}^{\circ }$ E. The energy of earthquakes was obtained from the JMA magnitude M after converting [57] to the moment magnitude M${}_{w}$ [43]. Setting a threshold M = 3.5 to assure data completeness, there exist 47,204 earthquakes in the area under discussion. Thus, we have on the average ∼150 earthquakes per month for the area considered.

The time evolution of the complexity measure $\Lambda_{i}$ is studied for a number of scales *i* of the seismicity with M ≥ 3.5 occurring in the whole area of Japan during the aforementioned 27-year period by choosing proper scales *i* as follows: We consider that recent investigations by means of natural time analysis showed that there exists the following interconnection between precursory low frequency (≤1 Hz) electric signals, termed Seismic Electric Signals (SES) (e.g., see Refs. [58,59]) and seismicity as follows [60]: The fluctuations β of the order parameter $\kappa_{1}$ of seismicity exhibit a minimum labeled $\beta_{min}$ when we observe the initiation of series of consecutive SES termed SES activities [51,61,62] exhibiting critical behavior [38,40,41] and have lead times ranging from a few weeks to around $5\frac{1}{2}$ months [11]. In addition, beyond this simultaneous appearance of SES activity and seismicity, Varotsos et al. [60] showed that these two phenomena are also linked closely in space, that gave the possibility of a reliable estimation of the epicentral area of an impending major earthquake. This has been subsequently confirmed in [35] for all major mainshocks of magnitude 7.6 or larger that occurred in Japan during 1984–2011 including the case of the M9 Tohoku earthquake. (Notably, before the latter earthquake, the minimum $\beta_{min}$ observed was the deepest during the whole period 1984–2011 investigated [56].) We return to this important point in Section 5. Before the initiation of the SES activity, and hence before $\beta_{min}$, a stage has been detected in which the temporal correlations between earthquake magnitudes exhibit an anti-correlated behavior [63] while after its initiation long range correlations prevail between earthquake magnitudes. Thus, a significant change in the temporal correlations between earthquake magnitudes occurs when comparing the two stages that correspond to the periods before and just after the initiation of an SES activity. Since this change may be captured by the time evolution of $\Delta S_{i}$, we start our investigation of $\Delta S_{i}$ from the scale of $i∼10^{3}$ events, which is of the order of the number of seismic events M ≥ 3.5 that occur during a period around the maximum lead time of SES activities.

## 4. Results

We first present the results of our investigation concerning the complexity measure $\Lambda_{i}$ associated with the fluctuations of the entropy change under time reversal.

**Figure 1:**
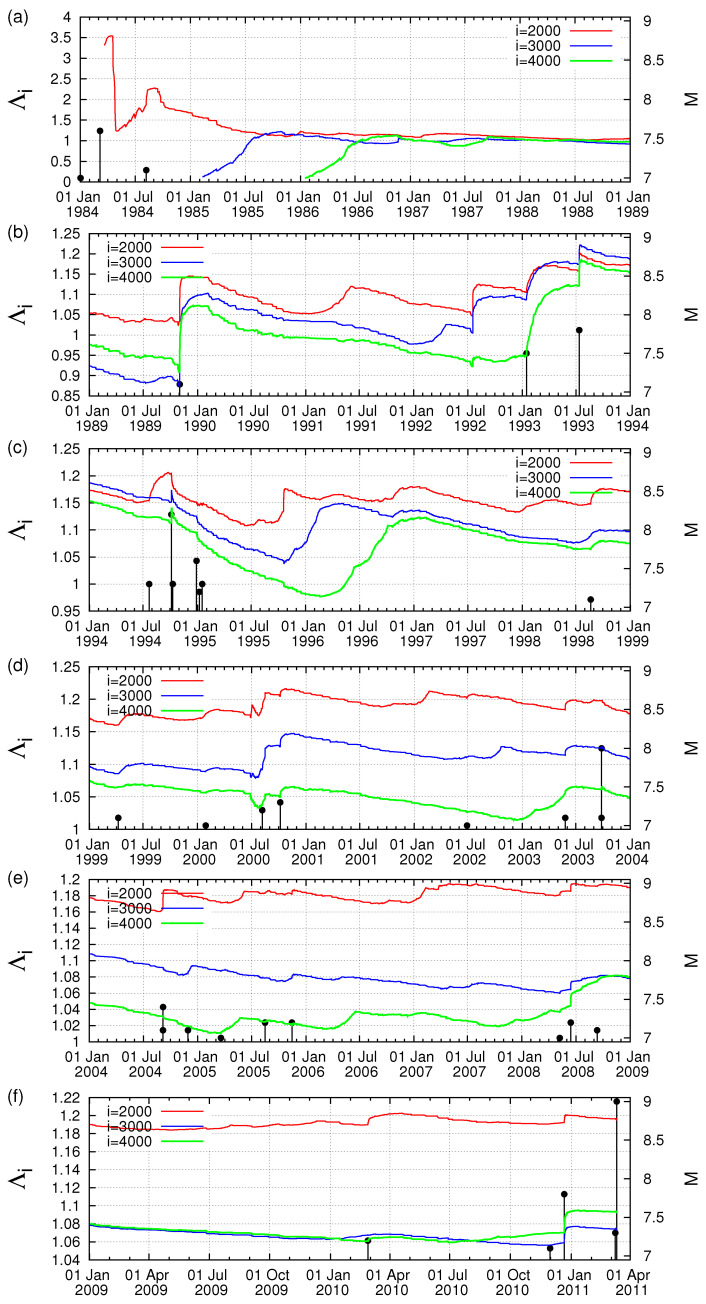
(**a**–**f**) Plot of the complexity measure $\Lambda_{i}$ versus the conventional time for the scales *i* = 2000 (red), 3000 (blue) and 4000 events (green) from 1 January 1984 until the M9 Tohoku earthquake on 11 March 2011. The vertical lines ending at circles depict the magnitudes (M ≥ 7) of earthquakes read in the right scale.

**Figure 2:**
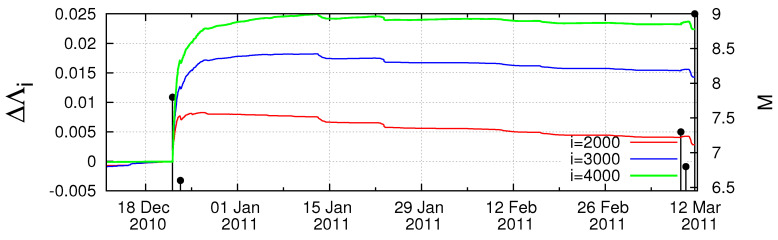
An almost three-month excerpt of Figure 1 in expanded time scale which shows the change $\Delta \Lambda_{i}$ of $\Lambda_{i}$ values versus the conventional time after the occurrence of the M7.8 earthquake on 22 December 2010 with an epicenter at 27.05${}^{\circ }$ N 143.94${}^{\circ }$ E. Note that, after the M7.3 foreshock that occurred on 9 March 2011, a decrease of the $\Delta \Lambda_{i}$ appears.

In Figure 1a–f, we plot the $\Lambda_{i}$ values for example for the time scales *i* = 2000, 3000 and 4000 events versus the conventional time from 1 January 1984 until the occurrence of the M9 Tohoku earthquake on 11 March 2011. In addition, all earthquakes of magnitude 7.0 or larger are also plotted in the same figure with vertical lines ending at circles read in the right scale. An inspection of this figure reveals that on 22 December 2010, i.e., almost two and a half months before the M9 earthquake an abrupt increase of the $\Lambda_{i}$ values for all the three scales is observed. This happens upon the occurrence of a M7.8 earthquake with an epicenter at 27.05${}^{\circ }$ N 143.94${}^{\circ }$ E [35]. This can be better visualized in Figure 2, which is a three-month excerpt of Figure 1f in expanded horizontal scale from 10 December 2010 to 11 May 2011 and depicts for the reader’s better inspection the increase $\Delta \Lambda_{i}$ of the $\Lambda_{i}$ values after the occurrence of the aforementioned M7.8 earthquake on 22 December 2010.

**Figure 3:**
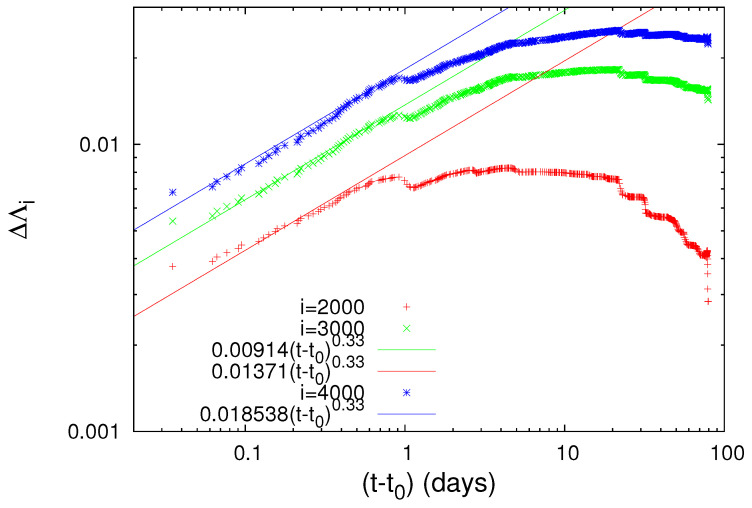
Log-log plot of the change $\Delta \Lambda_{i}$ of the complexity measures $\Lambda_{2000}$ (red), $\Lambda_{3000}$ (green) and $\Lambda_{4000}$ (blue) versus the elapsed time $\left(t-t_{0}\right)$ in days since the establishment of scaling behavior after the occurrence of the M7.8 earthquake on 22 December 2010. The value of $t_{0}$ is approximately 0.2 days measured from the M7.8 earthquake occurrence and c=1/3.

A close inspection of Figure 2 shows that all three complexity measures $\Lambda_{2000}$, $\Lambda_{3000}$ and $\Lambda_{4000}$ (cf. these symbols stand for the $\Lambda_{i}$ values at the scales *i* = 2000, 3000 and 4000, respectively) show a strong and abrupt increase on 22 December 2010 and after the occurrence of the aforementioned M7.8 earthquake exhibit a scaling behavior of the form

(6) $$\Delta \Lambda_{i}=A{\left(t-t_{0}\right)}^{c}$$

where the exponent *c* is independent of *i* with a value very close to 1/3, while the pre-factors *A* are proportional to *i* (see Figure 3) and $t_{0}$ is approximately 0.2 days after the M7.8 earthquake occurrence. Equation (6) conforms to the seminal work by Lifshitz and Slyozov [33] and independently by Wagner [34] on phase transitions which shows that the time growth of the characteristic size of the minority phase droplets grows with time as $t^{1/3}$.

**Figure 4:**
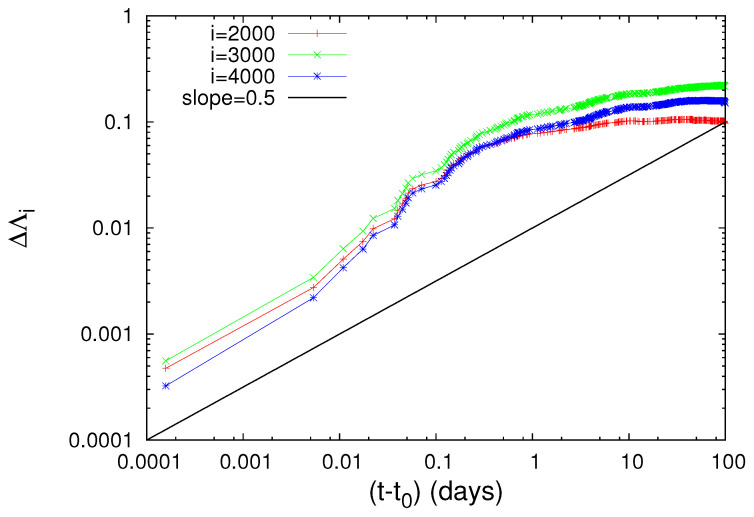
The same as Figure 3, but for the M7.1 earthquake on 2 November 1989 with an epicenter at 39.86${}^{\circ }$ N 143.05${}^{\circ }$ E. The value of $t_{0}$ is 0.022 days measured from the M7.1 earthquake occurrence and c=0.5.

**Figure 5:**
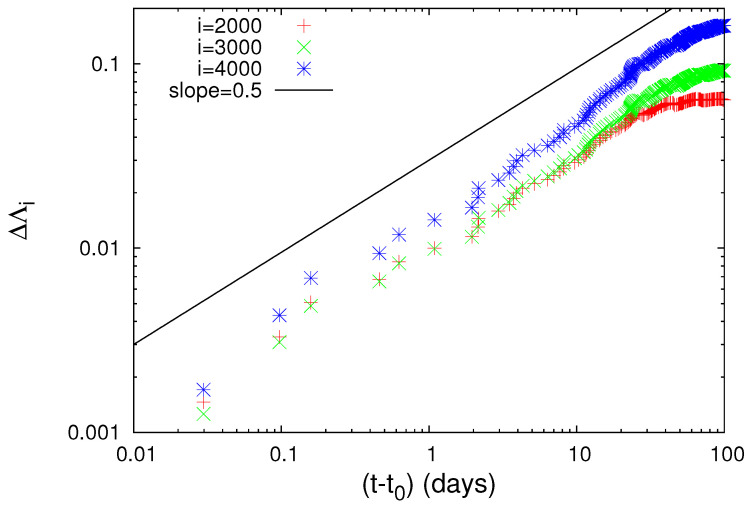
The same as Figure 3, but for the M7.5 earthquake on 15 January 1993 with an epicenter at 42.92${}^{\circ }$ N 144.35${}^{\circ }$ E. The value of $t_{0}$ is 0.014 days measured from the M7.5 earthquake occurrence and c=0.5.

To further elucidate the claim that an increase of $\Lambda_{i}$ is possibly associated with strong earthquakes, let us now further study the obvious increases (which are the most significant ones in Figure 1) of $\Lambda_{i}$ on 2 November 1989 and on 15 January 1993, as can be seen in Figure 1b, and investigate whether they exhibit a scaling behavior similar to that found above for the abrupt $\Lambda_{i}$ increase on 22 December 2010. Concerning the increase of $\Lambda_{i}$ on 2 November 1989, we give in Figure 4 the log-log plot of the changes $\Delta \Lambda_{i}$ of the complexity measures $\Lambda_{2000}$, $\Lambda_{3000}$ and $\Lambda_{4000}$ versus the elapsed time $\left(t-t_{0}\right)$ in days from the occurrence time of a M7.1 earthquake on the same date increased by 0.022 days. Furthermore, for readers’ convenience, we also plot in Figure 4 the black straight line of slope 0.5 in order to easily visualize that the value of *c* for the three complexity measures $\Lambda_{2000}$, $\Lambda_{3000}$ and $\Lambda_{4000}$ is close to 0.5, thus being distinctly different from the value c=1/3 predicted by the LSW theory. In addition, an inspection of Figure 4 reveals that the pre-factors are not proportional to *i* (e.g., see that for *i* = 3000 events the green line is higher than the blue line that corresponds to *i* = 4000 events). In other words, the increase of $\Lambda_{i}$ associated with the earthquake on 2 November 1989 does not obey Equation (6), thus strongly deviating from LSW theory of phase transitions. By the same token as in Figure 4, we now give in Figure 5 the log-log plot that corresponds to the changes $\Delta \Lambda_{i}$ of the complexity measures $\Lambda_{2000}$, $\Lambda_{3000}$ and $\Lambda_{4000}$ versus the elapsed time $\left(t-t_{0}\right)$ in days from the M7.5 earthquake occurrence on 15 January 1993 increased by 0.014 days. An inspection of this figure also reveals that the exponent in Figure 5 is close to 0.5—thus differing from the value c=1/3 of LSW theory—and that the prefactors *A* are not proportional to the scale *i*, for example see the red crosses corresponding to the scale *i* = 2000 which do not practically differ from the green symbols of the larger scale *i* = 3000.

**Figure 6:**
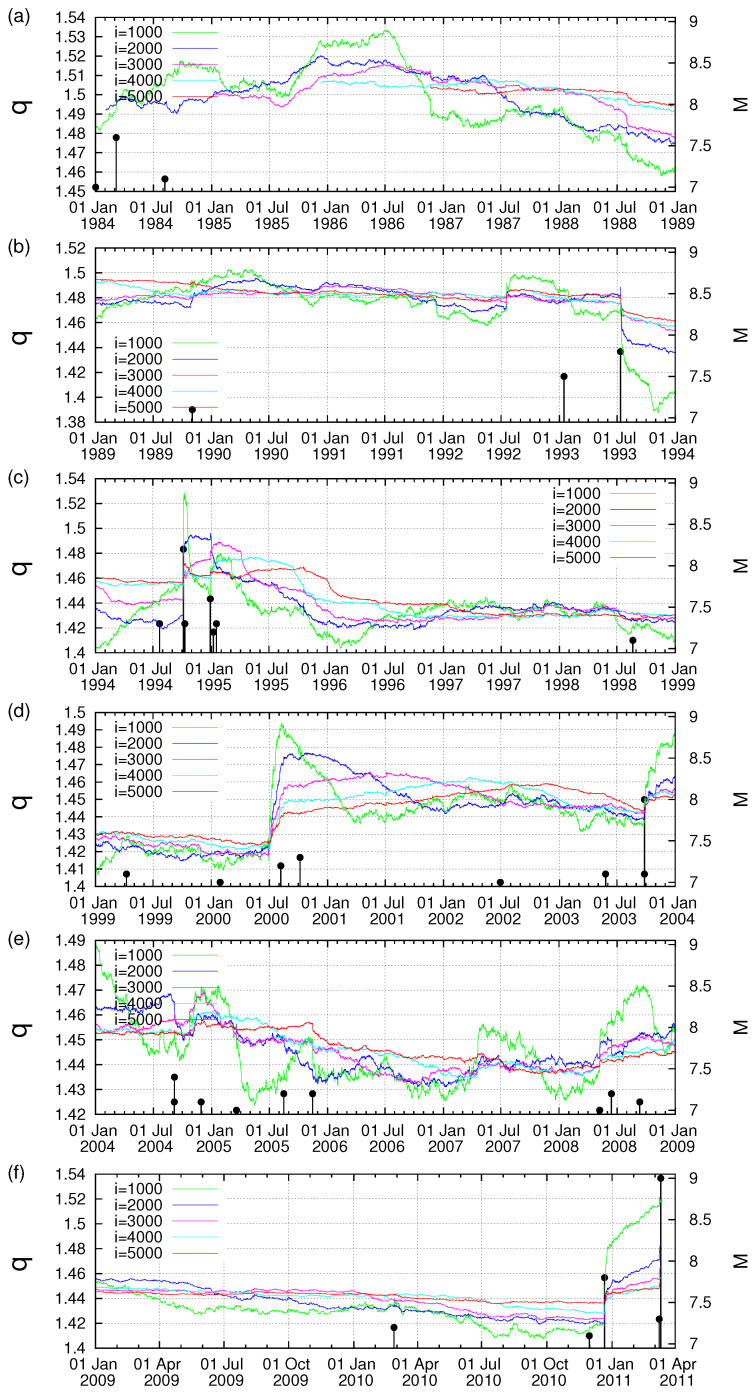
(**a**–**f**) Plots of the values of the Tsallis entropic index *q* at several scales *i* = 1000, 2000, 3000, 4000 and 5000; events as shown by the colors in the inset.

**Figure 7:**
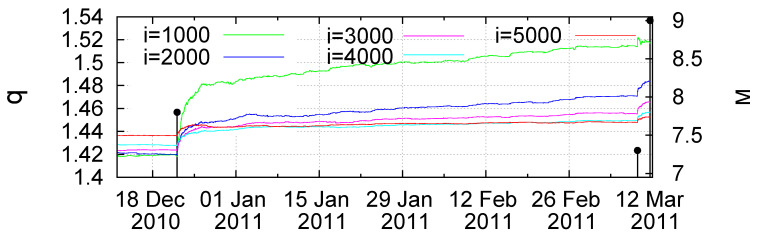
An almost three-month excerpt of Figure 6 after the occurrence of the M7.8 earthquake on 22 December 2010.

**Figure 8:**
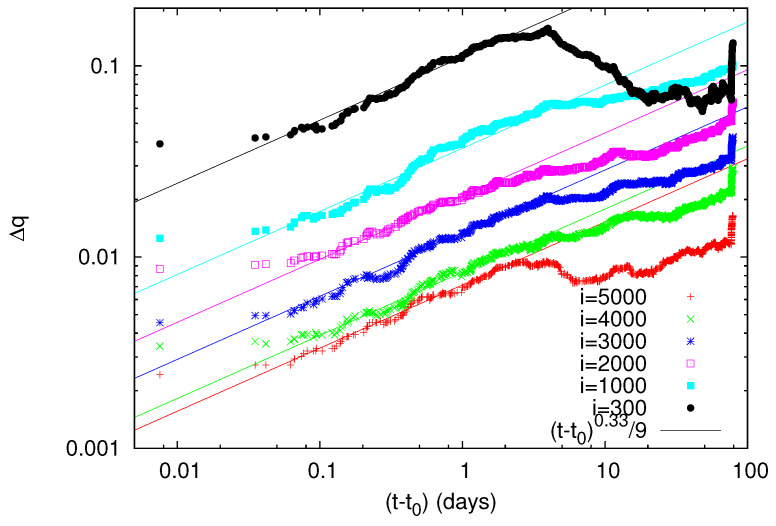
Log-log plot of the change Δq of the values of the Tsallis entropic index *q* versus the elapsed time $\left(t-t_{0}\right)$ in days since the establishment of scaling behavior after the occurrence of the M7.8 earthquake on 22 December 2010. The value of $t_{0}$ is 0.2 days measured from the M7.8 earthquake occurrence and c=1/3.

We now turn to the results obtained by means of non-extensive statistical mechanics [22], pioneered by Tsallis [19,24], which provides a framework for the study of complex systems in their non-equilibrium stationary states, systems with (multi)fractal and self-similar structures, long-range interacting systems, etc. This has found application [20,21] in the physics of earthquakes and especially in the description of the asperities in the faults on which earthquakes occur through the Tsallis entropic index *q*. Based on the earthquake magnitude distribution, one can deduce the [7,23] entropic index *q* and study how it varies with time as we approach a strong earthquake (for a recent review on this interesting aspect, see Ref. [32]). Figure 6 depicts the *q*-value versus conventional time during 1984–2011 as it is estimated [7] for several sliding windows of *i* = 1000, 2000, 3000, 4000 and 5000 consecutive earthquakes for M ≥ 3.5 in the Japanese area $N_{25}^{46}E_{125}^{148}$. We observe that, before the occurrence of the M9 Tohoku earthquake, the *q*-value exhibits an abrupt increase upon the occurrence of the M7.8 earthquake on 22 December 2010. A three-month excerpt of Figure 6 is given in expanded time scale in Figure 7 from 10 December 2010 until the Tohoku earthquake occurrence on 11 March 2011. By the same token as in Figure 3, Figure 4 and Figure 5, we now give in Figure 8 a log-log plot of the changes Δq of the Tsallis entropic index versus the time $\left(t-t_{0}\right)$ in days elapsed from the M7.8 earthquake occurrence on 22 December 2010 increased by approximately 0.2 days. An inspection of Figure 8 shows that the exponent *c* in Equation (6) is around 1/3, as in LSW theory, but the prefactors *A* are not proportional to *i*.

## 5. Discussion

A close inspection of Figure 2 reveals the following interesting feature of the complexity measure $\Lambda_{i}$: On 9 March 2011, a M7.3 foreshock occurred, and next day on 10 March 2011 all three complexity measures $\Lambda_{2000}$, $\Lambda_{3000}$ and $\Lambda_{4000}$ exhibited a simultaneous decrease. What is the origin of this decrease, since next day, i.e., on 11 March 2011, the M9.0 mainshock occurred?

**Figure 9:**
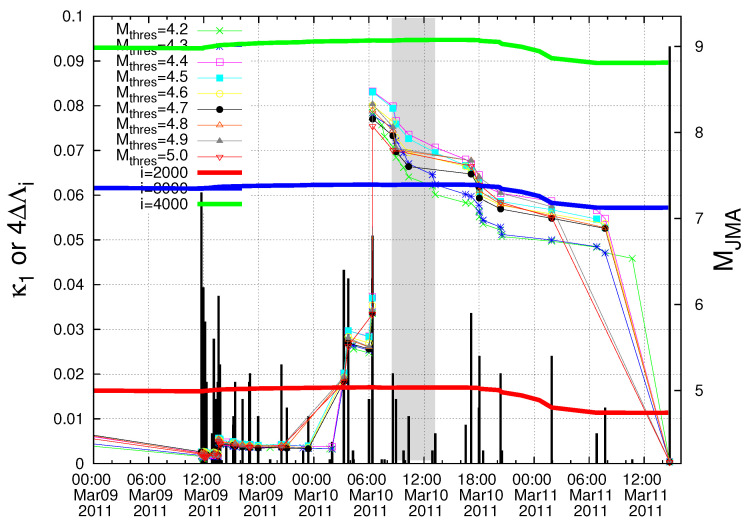
The $\kappa_{1}$ values as well as the values of the change $\Delta \Lambda_{2000}$, $\Delta \Lambda_{3000}$ and $\Delta \Lambda_{4000}$ of the complexity measures versus the conventional time since 00:00 LT on 9 March 2011 until the M9 Tohoku earthquake occurrence. The shaded area marks the period in the morning of 10 March 2011 during which the condition $\kappa_{1}=0.070$ is fulfilled.

The following comments are now in order, based on a recent review [64] on the procedure on identifying the occurrence time of an impending major earthquake by means of natural time analysis. This can be achieved by analyzing in natural time the earthquakes in the candidate area. To apply this procedure, we need two important pieces of information. First, we need to know when we should set the natural time equal to zero and start the analysis. This is the time at which the system enters the critical stage. Second, we need an estimation of the candidate epicentral area. If geoelectrical measurements are available, both pieces of information become available upon the recording of an SES activity, because its initiation marks the time when the system enters the critical stage, and in addition the SES data provide an estimation of the epicentral area of the impending mainshock. On the other hand, if geoelectrical data are lacking, we make use of the following two recent findings by means of natural time analysis: first, the fluctuations β of the order parameter of seismicity in a large area exhibit a minimum a few months before a major earthquake almost simultaneously with the initiation of an SES activity [60]; and, second, a spatiotemporal study of this minimum unveils an estimate of the epicentral area of the impending major earthquake [35]. The application of this procedure to the determination of the occurrence time of the M9 Tohoku earthquake by Varotsos et al. [64] was made as follows:

As for the starting time of the natural time analysis of seismicity, they chose the date of 5 January 2011 which is the date of the appearance of the minimum of the fluctuations of the order parameter of seismicity before this major earthquake reported by Sarlis et al. [56]. This, which remarkably is the deepest minimum ever observed during the period investigated (1984–2011), almost coincides with the initiation of an SES activity since anomalous magnetic field variations appeared in the vertical component during the period 4–14 January 2011 at two measuring sites (Esashi and Mizusawa) lying at epicentral distances of around 130 km [65,66,67]. As for the estimation of the epicentral location of the impending mainshock without making use of SES data, Sarlis et al. [35] worked as follows: By dividing the entire Japanese region N${}_{25}^{46}$E${}_{125}^{148}$ into small areas, a calculation of the fluctuations of $\kappa_{1}$ of seismicity is carried out on them. Some small areas show a minimum of the fluctuations almost simultaneously with the minimum in the entire Japanese region (on 5 January 2011) and such small areas cluster within a few hundred km from the actual epicenter, thus leading to an estimate of the candidate epicentral area. (Such an estimate of the epicentral area cannot be made of course in absence of the knowledge of the minimum of the fluctuations of the order parameter of seismicity on 5 January 2011. This absence happens as explained in the next paragraph if we do not consider the M7.8 earthquake on 22 December 2010.) A computation of the $\kappa_{1}$ values of seismicity in that area was made by starting from 5 January 2011. The results deduced by Varotsos et al. [64] are reproduced here in Figure 9 for $M_{thres}$ = 4.2 to 5.0. Recalling that at least six earthquakes are needed [10] for obtaining reliable $\kappa_{1}$ value, which happens in the candidate epicentral area on 16 February 2011 for $M_{thres}$ = 4.2, they depicted in their Figure 6a the computed $\kappa_{1}$ values during the last four weeks before the *M*9 Tohoku earthquake occurrence. This figure clearly showed that the condition $\kappa_{1}=0.070$ was not satisfied for all magnitude thresholds at least until the M7.3 earthquake on 9 March 2011. Here, we plot in expanded time scale in Figure 9 the $\kappa_{1}$ values of seismicity from 00:00 LT on 9 March 2011 until the Tohoku earthquake occurrence. This figure reveals that the condition $\kappa_{1}=0.070$ (which signals that the mainshock is going to occur within the next few days or so) is fulfilled for $M_{thres}$ = 4.2–5.0 in the morning of 10 March 2011 upon the occurrence of the earthquakes from 08:36 to 13:14 LT, i.e., almost one day before the Tohoku earthquake, see the gray shaded area in Figure 9. (Such a determination of the occurrence time of Tohoku earthquake cannot be achieved of course in absence of the knowledge of the date of the minimum of the fluctuations of the order parameter of seismicity on 5 January 2011, because from this date we started the computation as mentioned. This absence happens if we do not consider the M7.8 earthquake on 22 December 2010, as is explained in the next paragraph.) In this Figure, we have also inserted the values of the change $\Delta \Lambda_{2000}$, $\Delta \Lambda_{3000}$ and $\Delta \Lambda_{4000}$ (depicted in Figure 2) of the complexity measures but multiplied by a factor of four to better visualize their variations. Quite interestingly, we now clearly see that they exhibit a decrease just after the gray shaded area in Figure 9 where the condition $\kappa_{1}=0.070$ was fulfilled, thus signaling that the mainshock was approaching.

**Figure 10:**
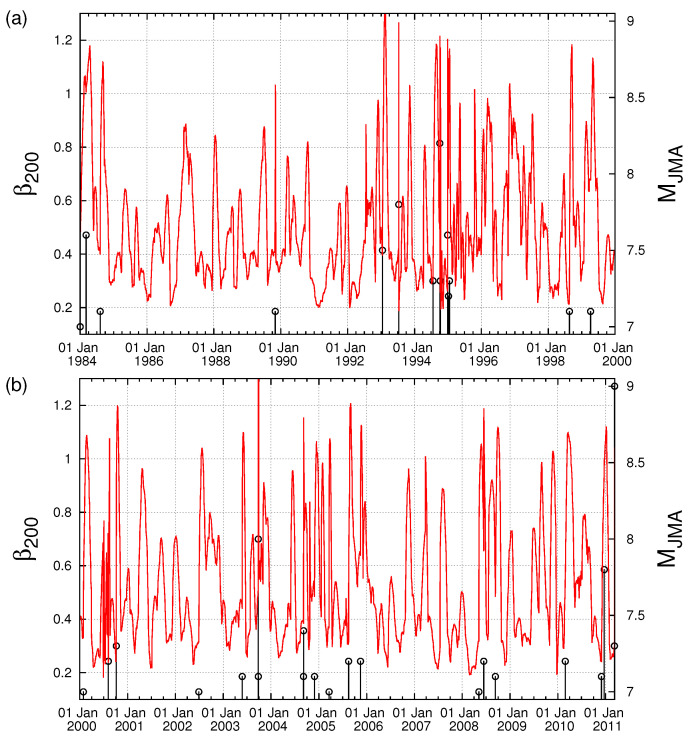
The fluctuations $\beta_{200}$ of the order parameter of seismicity when a window comprising 200 events is sliding through the JMA catalog (M ≥3.5) in the area N${}_{28}^{46}$E${}_{125}^{148}$ which does not contain the epicenter (27.05${}^{\circ }$ N,143.94${}^{\circ }$ E) of the M7.8 earthquake on 22 December 2010 for the periods: (**a**) 1 January 1984–1 January 2000; and (**b**) 1 January 2000 until the M9 Tohoku earthquake occurrence.

We now investigate what happens with the observation of the minimum of the fluctuations of the order parameter of seismicity if we do not consider the entire Japanese region N${}_{25}^{46}$E${}_{125}^{148}$ and select for example the area N${}_{28}^{46}$E${}_{125}^{148}$ which does not include the epicenter (27.05°N,143.94°E) of the M7.8 earthquake on 22 December 2010. Figure 10 depicts the fluctuations $\beta_{200}$ of the order parameter of seismicity when a natural time window comprising 200 events (M ≥3.5) is sliding through the JMA catalog of the area N${}_{28}^{46}$E${}_{125}^{148}$ from 1 January 1984 to the M9 Tohoku earthquake occurrence. An inspection of Figure 10 shows that the deepest minimum of $\beta_{200}$ during this period is not observed on 5 January 2010, as found in Refs. [42,56,63], when the investigation is extended to the entire Japanese region N${}_{25}^{46}$E${}_{125}^{148}$. The absence of this minimum reflects that neither the epicentral area nor the occurrence time of the M9 Tohoku earthquake can be estimated if we do not consider the M7.8 earthquake on 22 December 2010.

## 6. Main Conclusions

Almost two and a half months before the M9 Tohoku earthquake occurrence, i.e., on 22 December 2010, the following two facts were observed:

First, the complexity measure $\Lambda_{i}$ associated with the fluctuations of the entropy change of seismicity in natural time under time reversal exhibited an abrupt increase, which conforms to the Lifshitz–Slyozov–Wagner theory for phase transitions showing that the characteristic size of the minority phase droplets exhibits a scaling behavior in which time growth has the form $A{\left(t-t_{0}\right)}^{1/3}$ where the prefactors *A* are proportional to the scale *i*, while the exponent (1/3) is independent of *i*.

Second, the Tsallis entropic index *q* shows a simultaneous increase which interestingly exhibits the same exponent (1/3) (but the prefactors *A* are not proportional to the scale *i*).

Beyond the aforementioned two facts, we also found that the complexity measures $\Lambda_{2000}$, $\Lambda_{3000}$ and $\Lambda_{4000}$ exhibit a decrease just after the fulfillment of the condition $\kappa_{1}=0.070$ almost one day before the Tohoku earthquake occurrence.
